# Experience of non-operative management of blunt liver trauma at Hospital das Clínicas de Uberlândia: 114 cases

**DOI:** 10.1590/0100-6991e-20233424-en

**Published:** 2023-02-17

**Authors:** GABRIELA SOUZA FERNANDES, MURILO CÂNDIDO MARTINS, HEITOR LUIZ GOMES

**Affiliations:** 1 - Hospital das Clínicas da Universidade Federal de Uberlândia, Serviço de Cirurgia Geral - Uberlândia - MG - Brasil

**Keywords:** Advanced Trauma Life Support Care, Trauma Centers, Clinical Protocols, Liver, Cuidados de Suporte Avançado de Vida no Trauma, Centros de Traumatologia, Protocolos Clínicos, Fígado

## Abstract

**Introduction:**

trauma is the leading cause of death for the age group from 1 to 49 years in Brazil. Non-Operative Management (NOM) is the gold standard in trauma centers and does not affect mortality in comparison to operative treatment.

**Methods:**

medical records were reviewed for 114 patients with blunt liver trauma treated at Hospital das Clínicas of the Federal University of Uberlândia (HC-UFU) from November 2015 to November 2020.

**Results:**

the most prevalent gender was masculine (74.5%). The most prevalent age group was 20 to 49 years (65.7%). The majority of admitted patients (60.5%) had an Injury Severity Score (ISS) of more than 15. On hospital admission, 30.7% had HR above 100 bpm and 30.70% had SBP below 100mmHg. NOM was implemented in 77.2% of patients, the failure rate was 11.36% and the specific failure rate, excluding complications of associated injuries that resulted in surgery, was 1.75%. One third of deaths were due to severe traumatic brain injury.

**Conclusion:**

the failure rate of NOM in this study is similar to the literature reports for liver trauma. The failure rate, excluding complications of associated injuries, is considered low. The recognition of the epidemiological profile of patients admitted at HC-UFU allows multidisciplinary and integrated care with specialized training, as well as the development of institutional protocols, aiming to reduce morbidity and mortality related to hepatic trauma.

## RESUMO

**Introdução:**

o trauma é a principal causa de morte na faixa etária de 1 a 49 anos no Brasil. O Tratamento não operatório (TNO) é padrão ouro nos centros de trauma e não altera a mortalidade quando comparado ao tratamento operatório.

**Métodos:**

foram avaliados 114 prontuários médicos de pacientes vítimas de trauma hepático contuso atendidos no Hospital das Clínicas da Universidade Federal de Uberlândia (HC-UFU) no período de novembro de 2015 a novembro de 2020.

**Resultados:**

os homens foram o sexo mais acometido (74,5%) e a faixa etária mais prevalente foi de 20 a 49 anos (65,7%). 60,5% dos pacientes admitidos apresentavam um Injury Severity Score (ISS) maior ou igual a 16. Na admissão, 30,7% apresentaram FC acima de 100bpm e 30,70% PAS abaixo de 100mmHg. O TNO foi instituído em 77,2% dos pacientes, o índice de falha foi de 11,36% e o índice de falha específica, excluindo as cirurgias decorrentes por complicação de lesões associadas, foi de 1,75%. 33,33% dos óbitos foram decorrentes de traumatismo cranioencefálico grave.

**Conclusão:**

o índice de TNO instituído no HC-UFU é próximo ao estabelecido na literatura para traumas hepáticos. O índice de falha, quando excluídas às complicações por lesões associadas, é considerado baixo. O reconhecimento do perfil epidemiológico dos pacientes admitidos no HC-UFU permite o treinamento especializado integrativo e multiprofissional das equipes assistenciais e o desenvolvimento de protocolos institucionais, visando reduzir a morbimortalidade relacionada ao trauma hepático.

## INTRODUCTION

Trauma is the main cause of death in the age group of 1 to 49 years in Brazil^1^. The main mechanism is traffic accidents and about 5% of these victims’ hospitalizations are due to liver trauma^2^ ^,^ ^3^.

Victims of abdominal trauma who are hemodynamically stable and without signs of overt peritonitis on clinical examination are candidates for computed tomography (CT) scan of the abdomen with contrast^2^. According to the American Association for the Surgery of Trauma (AAST), liver injuries are classified in grades I to V, according to tomographic findings and severity, and non-operative management (NOM) may be instituted^4^ ^,^ ^5^.

NOM is the gold standard in trauma centers. It is responsible for reducing hospitalization time, abdominal infections, and the need for transfusion of blood components, without changing mortality when compared with surgical treatment^5^ ^-^ ^7^. There is evidence that the development of technologies and institutional protocols is associated with a reduction in trauma-related complications^2^.

## METHODS

This is a descriptive and observational study, in which retrospective data were collected from 114 medical records of trauma victims with the hospitalization diagnosis of “Traumatism of the liver or gallbladder” (ICD 10 D36.1), treated at the Hospital das Clínicas of the Federal University of Uberlândia (HC-UFU) from November 2015 to November 2020. Patients with penetrating trauma, admitted in cardiac arrest, without solid viscera injury, transferred from another service with insufficient data in the medical records, and with missing medical records were excluded from the study. The project was approved by the Ethics and Research Committee of UFU under number 43093520.9.0000.5152.

At HC-UFU, there is no institutional NOM protocol for liver trauma, and the decision on NOM is up to the on-duty surgeon. There is an Intensive Care Unit (ICU) for surgical patients, but not exclusively for trauma patients There is a hemodynamics service, but it does not work 24 hours a day. There is a radiology service, but emergency CT are not reported. Tomographic images can be discussed with the radiologist in case of diagnostic doubt on specific days and times during the weekdays. There is a Massive Transfusion Protocol (MTP) in the service, which is open in case of adult patients, victims of trauma, with ABC score ≥2 points, with suspected or confirmed bleeding, and clinical evidence of hemodynamic instability after the initial infusion of 1,000mL of crystalloid solution, pleural and/or pericardial decompression (when indicated), external bleeding control, pelvic bandaging and fracture alignment, and gastric and bladder emptying.

## RESULTS

Men were the most affected sex (74.5%). The most prevalent age group was the economically active, from 20 to 49 years old (65.7%). On admission, the Glasgow Coma Scale (GCS) was moderate or severe in 21.05% of patients and 60.5% of admitted patients had an Injury Severity Score (ISS) greater than or equal to 16 (Table 1).

**Table 1:** Demographic and severity characteristics of patients with blunt liver trauma admitted to HC-UFU from Nov 2015 to Nov 2020.

Demographic and severity characteristics	Number of patients
Sex	
Male	85
Female	29
Age	
<1 year	0
1-19 years	21
20-49 years old	75
50-79 years old	17
Demographic and severity characteristics	Number of patients
≥80 years	1
External cause	
Motorcycle accident	48
Bike accident	5
Car accident	29
Other types of vehicle accidents	2
Direct abdominal trauma	5
Fall from height	11
Fall from standing height	1
Run over	4
Assault	4
Burial	1
Others	4
GCS on admission	
≤8	19
9-12	5
13-15	90
SBP on admission	
SBP ≤100	35
SBP >100	77
Not recorded	2
HR on admission	
≤60 bpm	1
60-100 bpm	77
>100 bpm	35
Not recorded	1
ISS on admission	
1-15	45
16-24	32
25-75	37

**Table 2:** Non-operative treatment of blunt liver trauma. Classification of injuries by grade and their relationship with NOM failure.

Degree of injury	Number	Percentage	NOM failure
I	12	10.5%	0
II	28	24.5%	0
III	19	16.7%	4
IV	5	4.4%	1
V	0	0	0
Unclassified	32	28.1%	3
Others	18	15.8%	2

**Table 3:** Complications of NOM from blunt liver injury treated with surgery.

Type of injury	Number of patients
Liver-related complications	
Biliary fistula	1
Infected subcapsular hematoma	1
Associated injuries	
Splenic injury	5
Pancreatic trauma	3
Diaphragmatic hernia	1
Hollow viscera injury	1

On admission, 30.7% had heart rate (HR) above 100bpm and 30.70% had Systolic Blood Pressure (SBP) below 100mmHg. FAST was performed in 35.1% of the patients in the Trauma Room and was positive in 42.5% of these. The MTP was triggered in 14.9%, though only three (17.64%) of these patients were admitted with SBP ≤100mmHg, HR >100bpm, and positive FAST (Table 1).

Regarding the patients who underwent CT on admission, 28% of the injuries were not classified by the assistant team and 14% did not have an admission CT scan. As for the CT scans classified by the team, 18.7% corresponded to Grade I liver injury, 43.7% to Grade II, 29.7% to Grade III, and 7.8% Grade IV. There were no Grade V injuries.

NOM was instituted in 77.2% of patients admitted with liver trauma. The NOM failure rate was 11.36% (10), with only two resulting from liver-related complications: biliary fistula and infected subcapsular hematoma. In five of the failures there was associated splenic injury and in three of these the splenectomy was performed. The other patients were approached for other associated injuries: pancreatic trauma (three), diaphragmatic hernia (one), and hollow viscera injury (one).

Of the patients who were surgically approached immediately upon admission, that is, within 6 hours of trauma, 19.23% (five) underwent liver packing and 38.46% (10) underwent hepatorrhaphy or gelfoam placement as the main surgical procedure.

Of the total number of patients evaluated, 36.8% (42) were admitted to the ICU and 15.8% (18) died during hospitalization. Of these 18 deaths, 33.34% were due to complications of severe Traumatic Brain Injuries (TBI) and other 33.34% due to hemorrhagic shock (Table 4).

**Table 4:** Causes of death.

Cause of death	Number of patients
Abdominal trauma	2
Associated chest trauma	3
Hemorrhagic shock	6
Severe TBI	6
Sepsis	1

## DISCUSSION

Victims of blunt liver trauma are mostly from an economically active male population, in agreement with the results of national and international studies^8^ ^,^ ^9^.

In the present study, NOM was instituted in 77.2% of patients, a rate close to that established in the literature for liver trauma. However, in our service the NOM is instituted without a 24-hour radiology and hemodynamics center, considered by the literature as inclusion criteria to institute NOM. In addition, the rate of CT scans not reported by the attending physicians is high, which is another criticism of the inclusion of patients in the NOM criteria^10^.

The NOM failure rate of 11.36% is considered high when compared with other national studies. However, our study considered associated injuries and late complications, which evolved with surgical indication, such as NOM failure. Thus, if we consider the patients who evolved with surgical need liver-related complications, we have a specific failure rate of 1.75%, considering that the failures were mostly related to complications of associated injuries, such as spleen, pancreas, hollow viscera, and diaphragm. Furthermore, it is relevant to note that the profile of patients seen at the HC-UFU is mostly patients with multiple blunt trauma, with a high ISS in more than half of the patients evaluated in this study^11^.

More than a third of patients were admitted with hemodynamic instability and underwent FAST in the trauma room, with urgent blood transfusion being indication. However, in most patients receiving MTP, the institutional protocol was not properly followed.

We highlight the importance of the study in recognizing the epidemiological profile of patients admitted to the service to assist in the formulation of institutional protocols. Specialized training for polytrauma patients with multiple associated injuries and high risk of death from TBI should be integrative and multidisciplinary. Thus, we understand that the development, proper application, and follow-up of institutional protocols can optimize care and reduce morbidity and mortality associated with liver trauma in our service.
